# Floristic affinities of the lowland savannahs of Belize and southern Mexico

**DOI:** 10.3897/phytokeys.96.20097

**Published:** 2018-03-21

**Authors:** Idalia Arely Canché-Estrada, Juan Javier Ortiz-Díaz, Juan Tun-Garrido

**Affiliations:** 1 Departamento de Botanica, Campus de Ciencias Biológicas y Agropecuarias, Universidad Autónoma de Yucatán, carr. Mérida-Xmatkuil km 15.5, CP 97000, Yucatán, México

**Keywords:** Belize, biogeography, climate, México, relief, tropical grasslands

## Abstract

Environmental heterogeneity of Belize and southern Mexico savannahs as well as their geographical location suggest that these plant communities share floristic elements, making them conducive to a phytogeographical analysis. The aim of this study was to analyse the floristic affinities of nine savannahs of Belize and southern Mexico and to explain the similarities and differences amongst them.

A binary data matrix containing 915 species was built based on the authors’ own collections and on nine floristic lists already published. A second data matrix, consisting of 113 species representing trees, was also used since most literature on neotropical savannahs has focused on this life form. In addition, the ten most species-rich families as well as the characteristic species present in more than five savannahs were analysed. Floristic similarities were calculated using the Jaccard index. Dendrograms obtained in both types of analysis showed clusters with low similarity values, corresponding to geographic locations formed by the savannahs of Belize-Tabasco and the Yucatan Peninsula.

The floristic affinities of the savannahs may be explained in terms of heterogeneity in climate and physiography. The Yucatan Peninsula and Belize-Tabasco groups have differences in climate type and the amount of rainfall. In addition, the Yucatan Peninsula savannahs are established at the bottom of karstic valleys, while the Belize and Tabasco savannahs develop on extensive flatlands. The savannahs of Oaxaca have the same climate type and amount of rainfall as those of the Yucatan Peninsula but they are distributed along peaks and the slopes of shale hills. Fabaceae and Poaceae mainly dominated the local floras with 121 and 116 species each; remarkably, Melastomataceae was absent in the Yucatan Peninsula and Oaxaca. Nine species occurred in five to seven savannahs, confirming that they are widespread in both Belize and southern Mexico, and the Neotropics. Geographic location and floristic affinities of the nine savannahs support, to some extent, three different biogeographic provinces.

## Introduction

Savannahs cover approximately 40% of the area of the Neotropics (Furley 1999), occupying just over two million km^2^ (Mistry 2000). In Central America and the Caribbean, the savannah areas are not as extensive as, but they are ecologically similar to those in South America (Beard 1953, Huber 1987) and all neotropical savannahs are defined by their tendency to be on poorer soils and their grass-rich ground layer (Pennington et al. 2006). These plant communities are considered natural, but there also exist similar ones in anthropic areas, considered as secondary savannahs (Miranda and Hernández 1963, Pennington and Sarukhán 1968, 2005). Lowland savannahs generally experience annual events of waterlogging and drought related to the wet and dry seasons, so Sarmiento (1983) established a classification according to ecological criteria (i.e. seasonal and hyperseasonal).

Lowland savannahs of Belize and southern Mexico occur below 800 m and are defined here as any natural or semi-natural, fire-influenced ecosystem with a continuous herbaceous layer dominated by native grasses and sedges (Goodwin et al. 2013). Trees and shrubs may occur to a lesser or greater extent. Where present, *Pinus caribaea* Morelet (pine), *Acoelorrhaphe wrightii* H. Wendl. (palmetto), *Crescentia cujete* L. (calabash), *Byrsonima crassifolia* (L.) Kunth (craboo), *Curatella americana* L. (tachicon), Melastomataceae spp. and *Quercus oleoides* Schltdl. & Cham. (oak) are usually amongst the most structurally conspicuous non-herbaceous elements (Miranda and Hernández 1963, Rzedowski 1975, 1978, Bridgewater et al. 2002, Goodwin et al. 2013). Several plant associations (such as grass savannah, transitional savannah forest, gallery forests, oak savannah, palm savannah, pine savannah and wetland) have been described in Tabasco (Puig 1972) and Belize (Bridgewater et al. 2002, Penn et al. 2004, Stuart et al. 2006, Goodwin et al. 2013).

Gradients of floristic variation associated with latitude and longitude in neotropical savannahs have shown the great heterogeneity (Lenthall et al. 1999). In an analysis of the woody elements of neotropical savannahs, Lenthall et al. (1999) identified four phytogeographic zones: central Brazil and Bolivia extending to southern Amazonia; north of Amazonia extending across the isthmus of Central America and including the Caribbean; Belize, Guatemala and southern Mexico and north of the Mexican Plateau.

The savannahs of Belize and southern Mexico are floristically and environmentally similar, but such relationships have not been explored. Heterogeneity in climate and relief suggests gradients of floristic variation. Floristic knowledge, derived from several botanical surveys (Puig 1972, Pérez-García et al. 2001, Bridgewater et al. 2002, Farruggia et al 2008, Hicks et al. 2011, Várguez-Vázquez et al. 2012, Ortiz-Díaz et al. 2014), suggests that on a large scale they share floristic elements. However, on a local scale, they may differ.

The lowland savannahs of Belize and southern Mexico form an archipelago–like distribution and are thus discontinuous and embedded within other major vegetation types such as wet and seasonal forests as well as three biogeographic provinces, described by Morrone (2014). This archipielago-like distribution seems to be well suited for a phytogeographic analysis, so the aim of this study is: 1) to analyse the existence of common floristic patterns amongst Belize and southern Mexico lowland savannahs based on the authors’ own collections and floristic lists available in literature and 2) to describe the probable effects of physical factors on those floristic patterns.

## Methods

**Figure 1.**
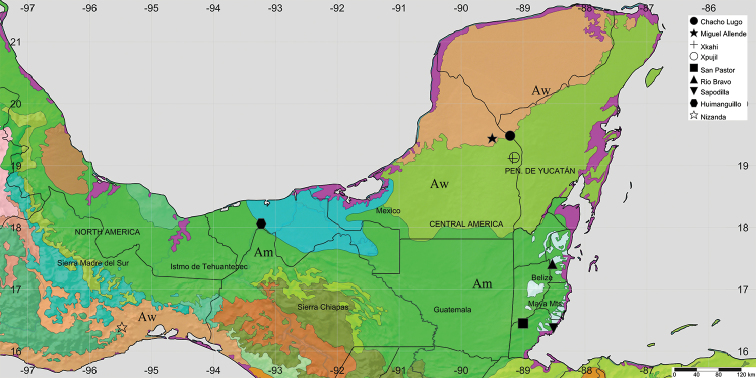
Geographic location of the nine savannahs of Belize and southern Mexico.

***Study area.***Nine sites of Belize and southern Mexico were selected to represent the lowlands savannahs as they have well documented floristic lists to compare with each other along the latitudinal gradient, as well as showing the heterogeneity in climate and physiography of three biogeographic provinces (Table 1) (Morrone 2014): four in the Yucatan Peninsula, four in Veracruz and one in the Pacific Lowlands. A map with the localities was built in SimpleMappr (Shorthouse 2010), according to the terrestrial ecoregions of the world (Fig. 1) (Olson et al. 2001). Terrestrial ecoregions exhibit a wide range of environmental conditions.

**Table 1.** Geographic location and environmental data of the lowland savannahs of Belize and southern Mexico. ^a^1: Ortiz-Díaz et al. (2014), 2: Várguez-Vázquez et al. (2012), 3: Hicks et al. (2011), 4: Bridgewater et al. (2002), 5: Farruggia et al. (2008), 6: Puig (1972), 7: Pérez-García et al. (2001).

Biogeographic Province	Site	Climate	Latitude N	Longitude W	Substrate	Altitude m.a.s.l	Mean annual temperature °C	Annual rainfall mm	^a^Source
Yucatan Peninsula	Chacho Lugo	Aw	19°48'N, 89°21'W		Clayey	77	26	1043	1
	Miguel Allende	Aw	19°44'N, 89°05'W		Clayey	90	26.3	1100	1
	Xkahi	Aw	19°11'N, 89°17'W		Clayey	135	26.3	1100	2
	Xpujil	Aw	19°13'N, 89°14'W		Clayey	92	26.3	1078	2
Veracruz	San Pastor	Am	16°45'N, 89°00'W		None	680	24.1	1500	3
	Rio Bravo	Am	17°41'N, 88°53'W		None	50	26–32	1500	4
	Sapodilla	Am	16°37'N, 88°51'W		Sand clayey	120	26	4526	5
	Huimanguillo	Am	18°06'N, 93°23'W		Sand clayey	50	26.2	2275	6
Pacific Lowlands	Nizanda	Aw	16°39'N, 95°48'W		Sand	160	25	1000	7

Regarding the climate, these savannah study areas can be grouped under the warm, humid tropical zone delineated by the Köppen system (García 1998) from Aw to Am. These ecoregions have a marked seasonality throughout the year; the dry season occurring from March to May and the rainy season with abundant rainfall during the summer (Table 1).

The geological substrate and geomorphic features along the Belize and southern Mexico region vary from north to south. In the north, the Yucatan Peninsula is formed mainly by a homogeneous limestone substrate with no rivers. In addition to limestone substrates, the middle portion of the study area belonging to Tabasco and Belize is more heterogeneous, being crossed by a complex fluvial system and including the Maya Mountains and the Highlands of Chiapas, which separate the regions from the Pacific Lowlands, where the Nizanda savannah is located (Table 1). The savannahs of the Yucatan Peninsula, Belize and Tabasco are developed at the bottom of karstic valleys (Várguez-Vázquez et al. 2012, Ortiz-Díaz et al. 2014), open flat areas (Puig 1972, Bridgewater 2002) and on slopes of the Maya Mountains (Penn et al. 2004, Hicks et al. 2011) while the Pacific lowlands savannahs are found close to or at the top of hills (Pérez-García et al. 2001).

***Data matrix construction***. The database was developed using the floristic lists already published for the study areas (Table 1) and collections made by the authors of this study in the Yucatan Peninsula. Due to some information being older than forty years, old scientific names were updated using TROPICOS (www.tropicos.org) and by following the classification system of the Angiosperm Phylogeny Group (Stevens 2001). All the species included in the published floristic lists were considered.

***Data analysis***. The flora compiled from the nine sites were compared using a multivariate method to obtain similarity measurements with the Jaccard coefficient and the UPGMA clustering technique. The matrix consisted of presence-absence data for 915 species. Since trees have been used as a model to assess the phytogeographical patterns in the neotropical savannahs (Lenthall et al. 1999), a data matrix was built with 113 tree species in order to gain more resolution and to explore how clusters change in relation to the full matrix. For both matrices, two analyses were performed for each matrix using all species and informative species only. The definition of groups was based on bootstrap values above 70 % after 5000 replicates were performed using the software PAST v.3.15 (Hammer et al. 2001). Analysis was also carried out on the number of species of the ten most species-rich families at each site in the form of a quantitative matrix to assess floristic relationships amongst the three biogeographic provinces proposed by Morrone (2014). The characteristic species of the savannah flora were determined by considering at least five sites where they were present.

## Results

**Figure 2.**
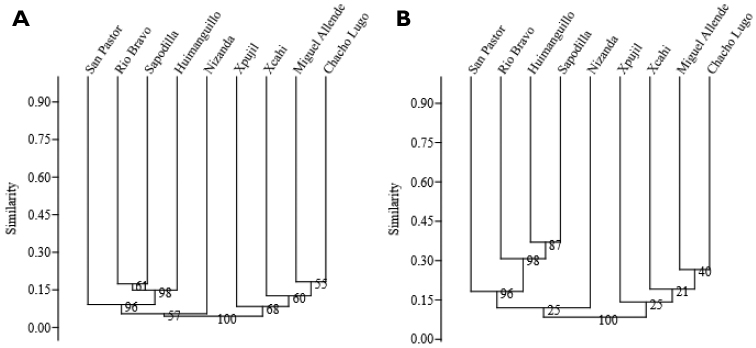
Similarity dendrograms for the nine savannahs of Belize and southern Mexico using 915 species. 2A: Built using all species. 2B: Built taking out single-site species.

**Figure 3.**
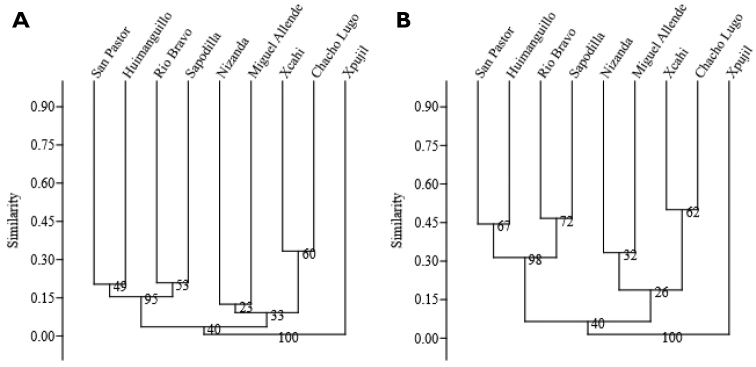
Similarity dendrograms for the nine savannahs of Belize and southern Mexico using 113 species of trees. 3A: Built using all species. 3B: Built taking out single-site species.

In general, dendrograms from all the anlyses conducted on the two matrices (full and trees only) showed clusters in a geographic arrangement and low similarity values amongst the nine sites (Figs 2, 3). Dendrograms 2A, 2B, using the whole flora and 3A and 3B, using the tree flora, grouped the Belize and Tabasco sites together with bootstrap values above 90 %. When Nizanda is grouped with this cluster, the bootstrap support is lower than 60 %. The four sites of the Yucatan Peninsula appeared clustered only when the whole species are considered but with a low bootstrap support. When the tree matrix is analysed (both considering the whole species and the informative species only), Nizanda’s savannah clusters with three sites of the Yucatan Peninsula savannahs, with a very low bootstrap support (Fig. 3A–B).

Fabaceae and Poaceae mainly dominated the flora of the studied sites with 121 and 116 species each. Table 2 shows the number of species for the ten most species-rich families of Belize and southern Mexico. These families sum up to 552 (60.32 %) species. Asteraceae, Euphorbiaceae, Convolvulaceae and Apocynaceae were absent in one site and remarkably, Melastomataceae was absent in five of the nine sites, four in the Yucatan Peninsula and one in Nizanda.

**Table 2.** The ten most species-rich families and number of species for each savannah.

	Chacho Lugo	Miguel Allende	Xkahi	Xpujil	San Pastor	Rio Bravo	Huimanguillo	Sapodilla	Nizanda	Total
Fabaceae	8	12	8	9	13	38	56	14	38	121
Poaceae	20	19	7	16	3	17	48	34	27	116
Cyperaceae	9	1	5	9	1	9	42	26	11	77
Asteraceae	4	0	2	3	9	8	27	2	14	60
Melastomataceae	0	0	0	0	12	7	24	9	0	34
Euphorbiaceae	1	1	5	0	4	2	5	17	1	33
Malvaceae	5	6	6	3	3	9	15	2	3	32
Rubiaceae	2	2	1	3	11	16	1	12	2	29
Convolvulaceae	6	5	6	3	0	4	8	2	3	27
Apocynaceae	0	1	1	3	2	6	7	2	5	23

Nine of 915 species were present in five or more sites, but none of the species was present in all sites. *Byrsonima crassifolia*, *Mimosa albida* Humb. & Bonpl. ex Willd. recorded at seven sites; *Piriqueta cistoides* (L.) Griseb., *Setaria parviflora* (Poir.) Kerguélen recorded at six sites; and *Andropogon virginicus* L., *Crescentia cujete, Paspalum plicatulum* Michx., *Psidium guineense* Sw. and *Sida linifolia* Juss. ex Cav. recorded at five sites were the widespread species of the lowland savannahs of Belize and southern Mexico.

## Discussion

Dendrograms of Belize and southern Mexico savannahs showed arrangements according to their geographical locations and suggested floristic patterns associated with heterogeneity in climate and physiography. Differences in the amount of annual rainfall and geomorphological characteristics may be the major determining variables at both continental and regional scales (Bailey 2009). The low floristic similarity amongst all sites (less than 50%) reflects the archipielago effect, since some of the studied sites are separated by extensive seasonal forests locally different in species composition (Pennington et al. 2006), rainforests (Puig 1972, Bridgewater et al. 2002, Pennington et al. 2006, Stuart et al. 2006) and xerophytic vegetation (Pérez-García et al. 2001, Pérez-García and Meave 2006).

The dendrograms resulting from the two anlyses were similar in structure except by the position of Nizanda and Xpujil savannahs. Both appeared as the most distant sites in the dendrograms and this may be explained by having more single-site species. Furthermore, the similarity levels increase when only shared species are taken into account, which reinforces the Belize-Tabasco and Yucatan Peninsula groups. The similarities and differences amongst clusters can be interpreted as a response to similar environmental conditions amongst some sites. In terms of climate, the Yucatan Peninsula savannahs are close to each other and share Aw climate, with relative low annual rainfall. Although the Nizanda savannah is distant on the Pacific Coast, it also has Aw climate. The Belize and Tabasco savannahs have Am climate with higher rainfall than the other regions. In terms of physiography, the savannahs of the Yucatan Peninsula are small and established at the bottom of karstic valleys (Várguez-Vázquez et al. 2012, Ortiz-Díaz et al. 2014), while the Belize and Tabasco savannahs develop on extensive flatlands (Puig 1972, Penn et al. 2004, Bridgewater et al. 2002). The savannahs on the Pacific Coast are distributed along peaks and the slopes of shale hills and this does not ocurr in other study regions (Rzedowski 1978, Pérez-García et al. 2001).

Plant inventories and differences in area size did not seem to influence the cluster outcome and lack of outstanding geological barriers may facilitate the similarity. In contrast, the archipelago effect may explain the low similarity values obtained at the adjacent sites of the Yucatan Peninsula. There, heterogeneity in physiographical and hydrological dynamics may lead to variation in the floristic composition (Ortiz-Díaz et al. 2014). The four Yucatan sites are categorised into two different geomorphological units; for instance, Miguel Allende is placed in an extensive karstic valley that favours rain overflow in the rainy season, while the other three savannahs are situated at the bottom of a tectonic valley forming part of a major structural valley system on a NW to SE orientation, such that they remain waterlogged during the rainy season (Várguez-Vázquez et al. 2012, Ortiz-Díaz et al. 2014).

Individual site floras were largely dominated by species of Fabaceae, Poaceae, Cyperaceae, Asteraceae, Melastomataceae, Euphorbiaceae, Malvaceae, Rubiaceae, Convolvulaceae and Apocynaceae. A similar floristic pattern was found in South American savannahs by Huber et al. (2006) in the Llanos of Venezuela and by Ratter et al. (2006) in the Cerrados of Brazil. It is possible that the relative abundance of the Melastomataceae family in the four savannahs of Belize (Penn et al. 2004) and Tabasco (Puig 1972) and its apparent absence in the Yucatan Peninsula and Nizanda is a good example of how the amount of rainfall defines Melastome distribution in Belize and southern Mexico since this family occurs in climates with high rainfall. High richness in Melastomataceae has been reported for other regions of tropical America in mesic, cool environments and on deep soils rich in nutrients, such as the Llanos of Venezuela (Huber et al. 2006) and the Cerrados of Brazil (Ratter et al. 2006).

The most common species of Belize and southern Mexico savannahs were the trees *Byrsonima crassifolia* and *Crescentia cujete*. Next most common, but only found in two or three localities were *Acoelorrhaphe wrightii*, *Curatella americana*, *Pinus caribaea* and *Quercus oleoides*. Along with these tree species, which are widesperead in neotropical grasslands, can be added the common shrubs *Mimosa albida*, and *Psidium guineense* and the herbs *Piriqueta cistoides*, *Setaria parviflora*, *Andropogon virginicus*, *Paspalum plicatulum* and *Sida linifolia*, which also shape the so-called basic floristic matrix present in most of neotropical savannahs (Huber 1987, Lenthall et al. 1999, Bridgewater et al. 2002). Floristic affinity of the common species confirms that the geographic origin of savannah flora of the study region is mainly neotropical.

Geographic location and floristic affinities of these nine savannahs support to some extent three different biogeographic provinces, proposed by Morrone (2014) as follows: four savannahs in the Yucatan Peninsula, four in Veracruz and one in the Pacific Lowlands. Moreover, the nine savannahs show the influence of environment associated with heterogeneity in the climate and physiography of these plant communities.

Lastly, it is emphasised that the floristic variation amongst these savannahs and their unique contribution to the flora of Central America give them high conservation importance. Yet they are environmentally undervaluated and severely affected by human activities. Conserving Central American savannahs is more of an urgent task for Mexico, since the Belize conservation system cover savannah areas (Bridgewater et al. 2002, Hicks et al. 2011).
